# A prospective observational study on the beneficial effects and tolerability of a cetylated fatty acids (CFA) complex in a patch formulation for shoulder tendon disorders

**DOI:** 10.1186/s12891-022-05304-x

**Published:** 2022-04-12

**Authors:** Rosaria Lanzisera, Alessandro Baroni, Gaetana Lenti, Elisabetta Geri

**Affiliations:** UOC Recupero E Riabilitazione Funzionale, Ambito Territoriale Pisano, ASL Toscana Nordovest, Pisa, Italy

**Keywords:** Tendinopathy, CFA, Constant Murley Score

## Abstract

**Background:**

The advancement of physiopathological knowledge of tendon structures has shown that, in conditions of overload, there is the onset of both degenerative phenomena, such as the production of metalloproteases, apoptosis of tendon cells and neoangiogenesis, and regenerative and protective phenomena, such as the production of IGF-1 and nitric oxide. Tendinopathy results from the imbalance between these two groups of factors, leading to degeneration, weakening, and fissuring of the tendons, with the presence of local pain. The aim of the study was to evaluate the efficacy and tolerability of cetylated fatty acids (CFA) patch formulation in the control of acute localized shoulder pain and recovery of function in patients with tendinopathies.

**Methods:**

A prospective, single-center, no-profit observational study conducted in accordance with Good Clinical Practice. Thirty patients with recent onset shoulder pain symptoms (1–3 months) related to bursitis and tenosynovitis, with a diagnosis of shoulder tendon pathology confirmed by ultrasound examination, was evaluated for shoulder pain and function using the Constant Murley Score. Patients used 1 patch containing CFA for 8 h per day for 10 days. At 10 (V1) and 35 (V2) days after the first visit (V0), the Constant Score, treatment compliance and product tolerability were evaluated.

**Results:**

Thirty patients completed the treatment. At V0 the mean Constant Score (CS) was 32.37 ± 11.86, during V1 the mean CS was 50.68 ± 14.30, and at V3 the mean CS was 51.07 ± 15.29. The CS increased significantly between V0 and V1 (*p* < 0.0001) but did not vary significantly between V1 and V2 (*p* = 1). The tolerability of the product was excellent.

**Conclusions:**

Application of the CFA-based patch for 10 consecutive days in patients with shoulder tendinopathies was effective in reducing local pain and resulted in a good recovery of function. The results achieved at day 10 were maintained for 25 days, until the follow-up visit at day 35. CFA-based patch, thanks to their efficacy and tolerability, seems to be a promising solution to improve pain and functionality in subject with shoulder tendinopathy.

**Trial registration:**

The study was approved by the Ethics Committee of Azienda USL Toscana Nord Ovest (protocol code 2018RIAB105) and conducted in accordance with Good Clinical Practice and the ethical principles outlined in the Declaration of Helsinki.

## Introduction

The advancement of knowledge on tendon physiopathology has shown that in conditions of overload, probably associated with oxidative stress mechanisms, happens both degenerative and regenerative and protective phenomena. Degenerative processes include the production of metalloproteases that degrade the extracellular matrix, apoptosis of the tendon cells and neoangiogenesis with vascular infiltration. The regenerative and protective phenomena include the production of IGF-1 (insulin-like growth factor-1) and nitric oxide. Tendinopathy is the result of an imbalance between these two sets of factors, leading to degeneration, weakening, and fissuring of the tendon, associated with local pain [1].

Currently, the treatment of tendinopathy involves the oral administration of NSAIDs, corticosteroid infiltrations, physical therapy (laser and/or ultrasound,) and shock waves (Extracorporeal Shock Wave Therapy). These therapies can have some limitations: 1) the available literature suggests that in absence of an ongoing inflammatory process, NSAIDs are not able to modify the course of chronic tendinopathy [2–4] ; 2) corticosteroid infiltrations improves the symptoms in the majority of patients, but numerous cases of tendon rupture have been reported [5–7]; 3) shock waves, recently becomed popular to treat soft tissue disorders, have given ambiguous results in the latest published evidences mainly due to different protocols applied [8, 9].

In this context, it is important to emphasize that numerous studies have shown the effectiveness of topically administered cetylated fatty acids (CFA) in improving joints mobility, functionality, strength and endurance, as well as reducing pain symptoms in absence of side effects [10–14]. CFA have been shown to play a role in synovial membrane protection and cell membrane stabilization, promoting normal flexibility and mobility, leading to a reduction in pain and an increase in joint fluidity and lubrication [15]. In a recent publication, the combination of CFA with conservative rehabilitation therapy improved muscle strength, reduced pain and accelerated the recovery process in professional hockey players [16]. The same CFA have also been used for the topical treatment of knee osteoarthritis, resulting in reduced pain and consequently improved mobility [12]. However, there is no available study dealing with CFA for shoulder tendinopathy.

The aim of this study was to evaluate the efficacy and tolerability of CFA patch formulation in controlling acute localized shoulder pain and recovery of function in patients with tendinopathies.

## Methods

This was a prospective, single-center, no-profit, observational study conducted at the Department of Rehabilitation of the Azienda Usl Toscana Nord Ovest (Italy). The study was approved by the local Ethics Committee (protocol code 2018RIAB105) and conducted in accordance with Good Clinical Practice and the ethical principles outlined in the Declaration of Helsinki. All patients signed the informed consent document.

### Partecipants

Patients referred to the center between 1 October 2019 and 31 July 2020 who had suffered from recent onset (1–3 months) shoulder pain symptoms related to bursitis and tenosynovitis, with a diagnosis of shoulder tendon pathology confirmed by ultrasound examination, were included. The exclusion criteria were the following: previous treatment with NSAIDs or other self-medication products or instrumental techniques for their tendinopathy; known hypersensitivity to CFA or to one of the product excipients, or injured skin in the shoulder area; use of NSAIDs and corticosteroid infiltrations during treatment; cognitive impairment or full-thickness rotator cuff tendon tear.

### Study design

**Fig. 1:**
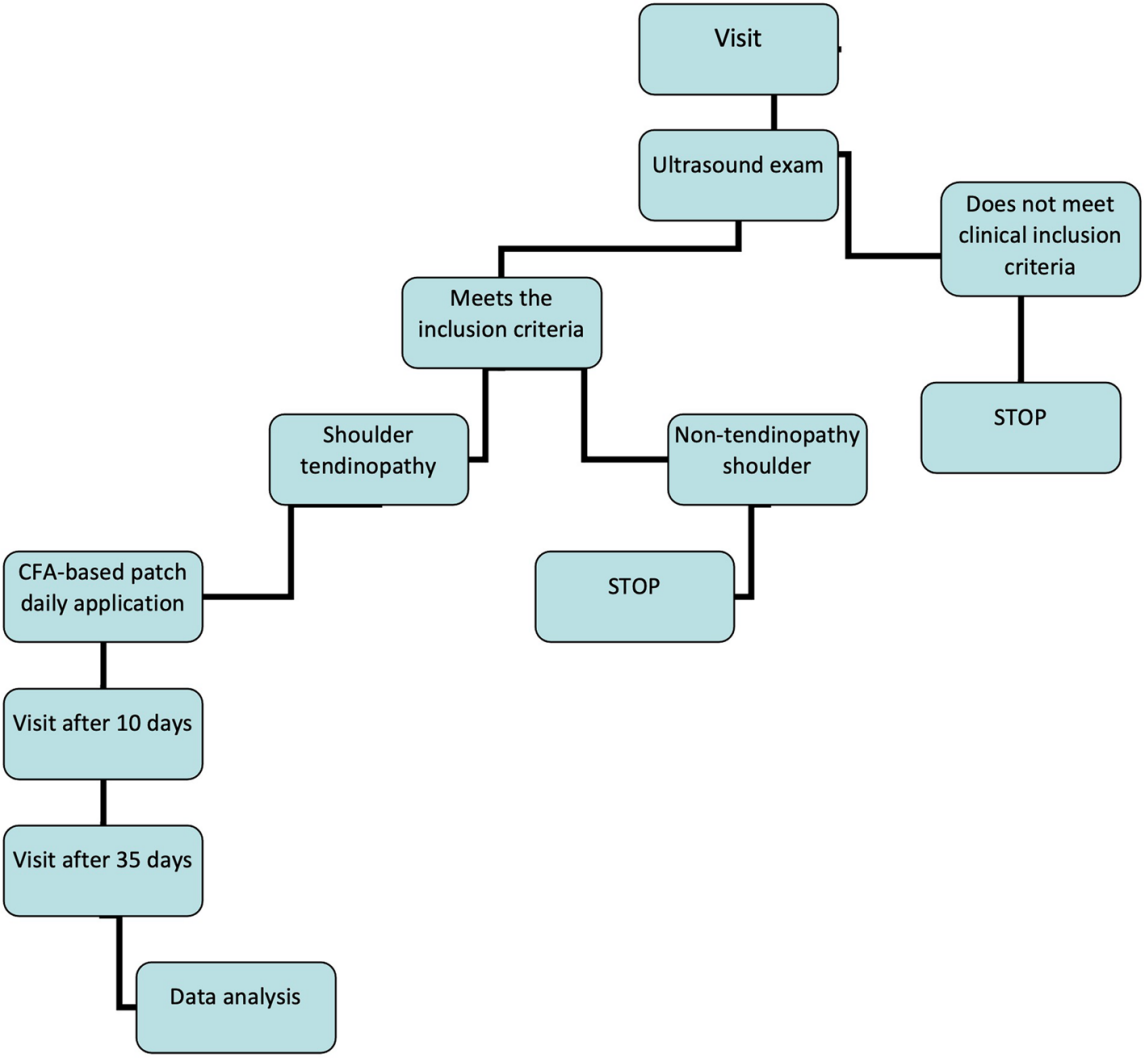
Flow chart of the study

At the first visit (V0) the patients were examined by ultrasound examination, and pain and shoulder function were assessed using the Constant-Murley Score (CS). All patients were prescribed to use at home 1 patch containing CFA (Cetilar® Patch, Pharmanutra SpA, Pisa, Italy) for 8 h a day for 10 consecutive days. The clinician indicated where to apply the patch according to the painful area. The CFA-based patch were used for 8 consecutive hours a day to allow the active ingredient to be fully released though the skin, in accordance with the instructions for use indicated by the manufacturer. Two follow-up visits were carried out: the first (V1) 10 days after the first visit corresponding to the treatment period, the second (V2) 35 days after the first visit (Fig. 1 shows the flow chart of the study). At each visit, Constant Score, treatment compliance and product tolerability (via the recording of any undesirable effects) were assessed.

### Assessment

The CS is a 100-point scale that defines the patient's level of pain and ability to perform normal everyday activities. This score is often used to determine functionality after treatment of a shoulder injury. The test is divided into 4 subscales: two subjective: pain (15 points) and activities of daily living (ADL) (20 points) and two objective: range of motion (ROM) (40 points) and strength (25 points). The maximum achievable score (100) represents an optimal condition [17].

### Salmple sizing

The sample size was based on the primary endpoint, by assessing the efficacy of CFA patch formulation in controlling acute localized shoulder pain in patients with tendinopathies. A variation of at least 3 points in the Constant Score was estimated, setting a type error α = 0.05 and taking as a starting point a test power (β) of 0.9 (90%), resulting in a sample size of at least 30 subjects in order to ensure adequate statistical robustness of the study.

### Statistical analysis

The normality of the distributions of continuous variables was assessed using the Lilliefors corrected Kolmogorov–Smirnov test. Accordingly, continuous variables were represented as mean ± standard deviation or median and interquartile interval, depending on their distributions. Independently of the distributions, given the small sample size, all comparisons between groups were performed with non-parametric Mann–Whitney test, for unpaired comparisons, and Wilcoxon test, for paired comparisons. The differences among continuous variables on different time points were assessed with Bonferroni corrected Wilcoxon test. In all cases, a *p*-value below 0.05 was considered significant. The statistical analysis was carried out using Excel 2020 (Microsoft, Redmond, Washington) and GraphPad Prism version 8.3.1 (GraphPad Software, San Diego, California).

## Results

A total of 32 patients were recruited. Thirty patients completed the study. One patient failed to complete the treatment due to an irritative reaction not product-related, another was unable to return to the follow-up due to an accident (bone fracture) during the observation period. Table 1 shows the characteristics of the patients enrolled.

**Table 1:** Study population

Characteristics	All	Males	Females
Number	30	11 (36.7%)	19 (63.3%)
Age	60.1 ± 10.3	61.9 ± 9.1	59.0 ± 11.0
Shoulder with tendinopathy	18 right/12 left	5 right/6 left	13 right/6 left
Dominant shoulder	19	6	13

The sample was mainly represented by women (19 vs 11 men); most of the participants in the study had dominant shoulder pain. There were no significant differences in the mean age between the two groups (*p* = 0.3198).

### Analysis of the entire sample

At V0 the mean Constant Score (CS) was 32.37 ± 11.86. At the second visit (V1), performed on average 12 days after the first one, the mean CS value was 50.68 ± 14.30. Ate the tirth follow-up visit (V2), performed on average 35 days after the first visit, the mean CS value was 51.07 ± 15.29. The single indices values constituting the CS for each timepoint are reported in the Table 2.

**Table 2:** Description of single values constituting the Constant Score for each timepoint

	**V0**	**V1**	**V2**
**Pain**	3.3 ± 2.76	7.65 ± 3.78	7.6 ± 3.4
**Activities of daily living (ADL)**	6 [6 —10]	16 [12.5—20]	16 [11.25—19.5]
**Range of motion (ROM)**	17 [14—20]	22 [18.5—31]	25 [20—32]
**Strength**	3 [1.25—3]	3 [3—3.75]	3 [3—3.75]

**Fig. 2:**
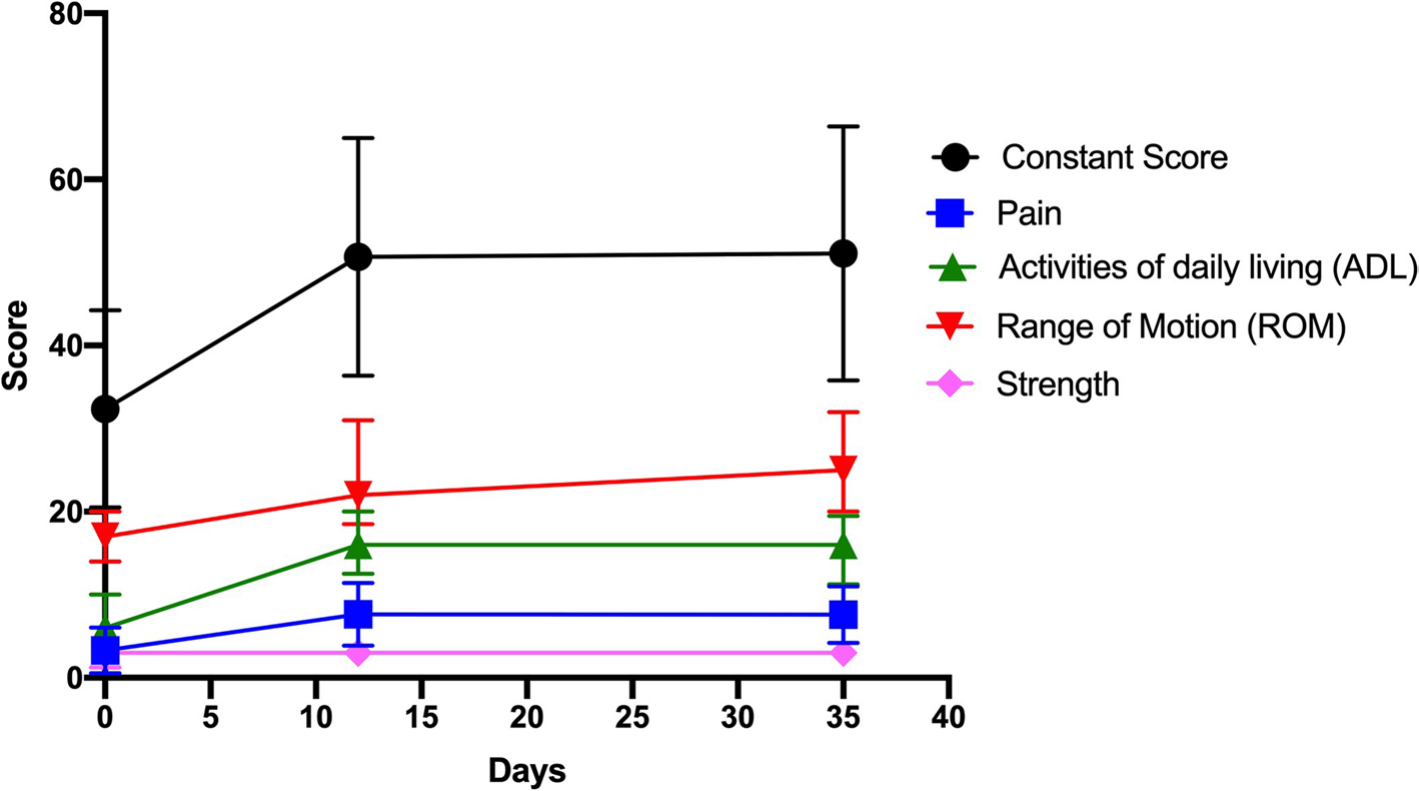
Constant Score with graphical representation of all its indices. Continues variables (CS and Pain) are graphically represented as mean and standard deviation. Not continues variables (ADL, ROM and strength) are graphically represented as median and the error bar represent the interquartile range

The CS value increased significantly between V0 and V1 (*p* < 0.0001), whereas it was steady between V1 and V2 (*p* = 1). Pain, ADL, and ROM followed the same trend as the total score. Strength did not change significantly between V0 and V1 ( *p* = 0.5208), neither between V1 and V2 (*p* = 1). The CS values at different time-points are shown in Fig. 2.

**Fig. 3:**
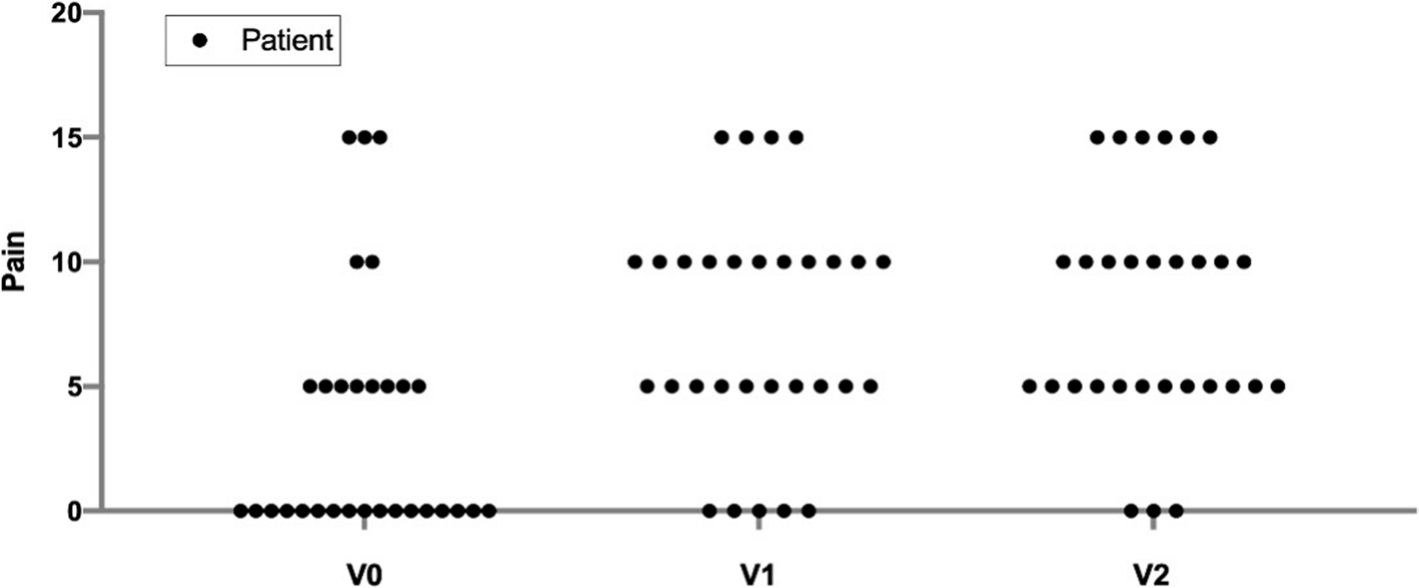
Patients’ response to question A1 related to frequency of pain

By analyzing each parameter constituting the CS, it emerges that 17 patients (56.7%) reported a score of 0 to the first question of the section “Pain” (A1) at V1, which on a pain frequency scale represents "permanent" pain without forcing; among these, only 5 (16.7%) still suffered from "permanent" pain at V1 and only 3 (10%) at V2 (Fig. 3).

**Fig. 4:**
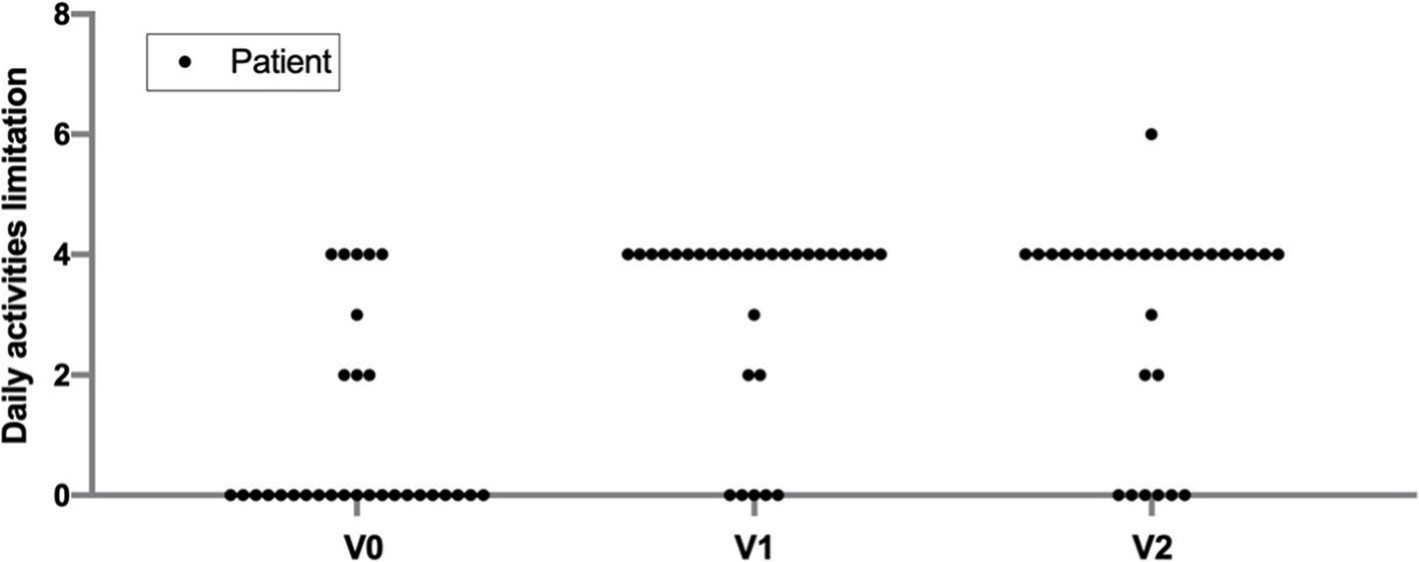
Patients’ response to question B1 related to the limitation of activities of daily living

If we analyze the first question of the section “activities of daily living” (B1), which evaluates the patients' limitation for daily activities, it can be observed how 21 patients (70%) reported a severe limitation of their work or daily activities at the first visit, while only 5 (16.7%) reported the same limitation at V1 (Fig. 4).

The tolerability of the product was excellent: only 1 case of skin irritation was reported with the use of the patch and, although the irritation was temporary and resolved spontaneously indicating a non-product related effect, the patient preferred not to continue with the study.

### Constant Score improvement percentage

**Fig. 5:**
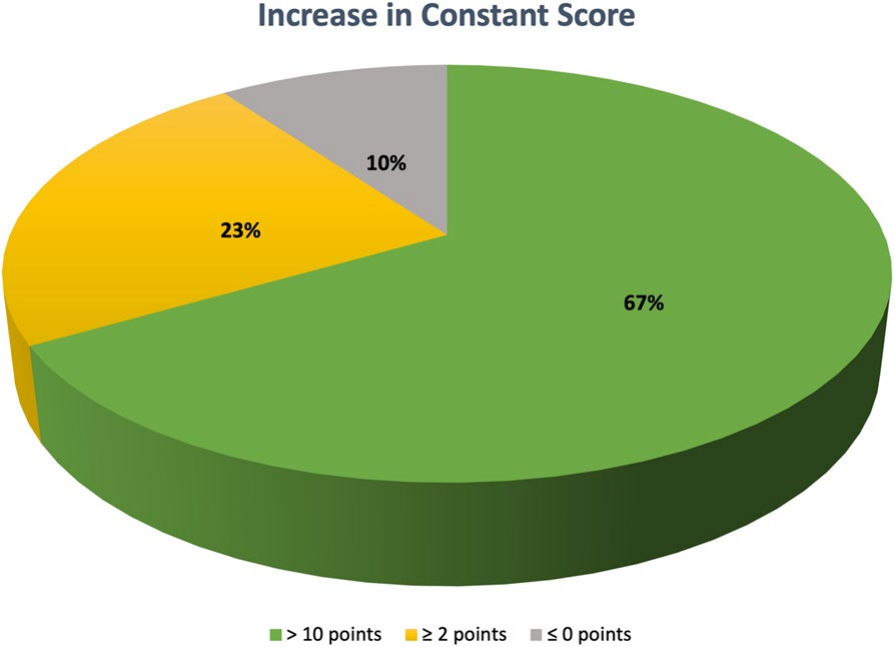
Percentage of patients reporting changes in CS

Constant Score values increased by 10 points or more in 20 patients (66.7%), between 2 and 10 points in 7 patients (23,3%), not changed in 2 patients (6,7%), and decreased in 1 patient (3,3%). (Fig. 5).

**Fig. 6:**
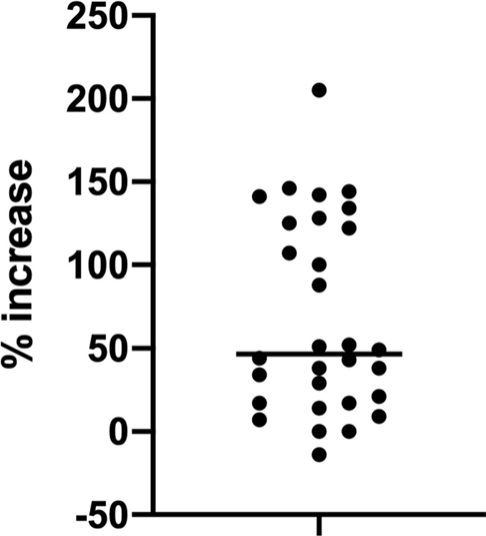
Percentage increase in CS between V0 and V1. Each point represents one patient

In percentage there was an average increase in the CS value of 68% between V1 vs V0. Only 3 patients (10%) failed to report an increase in CS value, while 14 patients (46.7%) reported an increase of more than 50% (Fig. 6).

Between V2 and V1, almost all patients reported no significant change in the CS value, except for three patients who reported a high increase in the CS value at V1.

## Discussion

Different approaches to the treatment of pain due to shoulder tendinopathies are reported in literature. In a recent review of currente literature, Pieters et al. suggests the exercise therapy as first-line treatment to improve pain, mobility and function, possibly in combination with manual therapy. Discordant evidence is reported regarding the use of multimodal physiotherapy or corticosteroid injections. Ultrasound, laser therapy and shock waves show little evidence of efficacy [18]. According to the clinical experience of the authors of the present study, shoulder tendinopathies have a complex clinical course: pain and function do not improve without any type of treatment, with a tendency to become chronic. As far as we know, this is the first study to date showing the usefulness of topical CFA in reducing pain, and improving mobility and function in shoulder tendinopathies. CFAs are esterified fatty acids from vegetable origin characterized by a rapid absorption following topical administration. CFAs can easily permeate the skin through a passive chemical-physical gradient, favored by the lipid nature of cell membranes [19]. CFAs acts by protecting the synovial membranes and by stabilizing the cell membranes. These actions allowing for normal flexibility and mobility of joints, and resulting in a reduction of pain and increased fluid at joints level, which contributes to their normal lubrication [15, 20]. Kraemer et al. were the firsts to evaluate the application of a CFA-based cream to reduce pain and improve function in patients with knee osteoarthritis [13, 14]. Subsequently, Ariani et al. also demonstrated that the application of a cream containing the same CFA complex used in this study reduced pain in patients with knee osteoarthritis and improved function following assessment with the WOMAC questionnaire [12]. Pampaloni et al. evaluated the application of a patch containing CFAs in a combined protocol with manual therapies, and demonstrated that was useful in roller hockey athletes in reducing pain and improving muscle function [16].

In the present’ study, the effect of CFA patch formulation in reducing pain and improving function in patients with shoulder tendinopathy was evaluated. The outcomes were assessed after a 10-day period of application of the preparation and each patient was re-evaluated at a follow-up 35 days after the first visit. The choice of a 10-day period of application ad 35 days for the follow-up visit was in line with in normal clinical practice and it is useful to demonstrate the effects of treatment in a relatively short period and to check the patient's progress approximately one month after diagnosis. The most important finding was that, after the treatment period, the CS value significantly increased in 87% of the subjects, witout the use of physical therapies or use of corticosteroids for systemic or local use.

Although there was some variability depending on the sex and age of the subjects examined, in this study all patients started with a very low average CS value (32.37 ± 11.86), considering that healthy subjects of the same age and sex should have a CS ranging between 70 and 90 points [21, 22].

After the first treatment period of 10 days, the CS value stabilized for the following 25 days, and approximately the same value was reported at the follow-up visit on day 35. This could be explained by the fact that the 10-day treatment is a time period appropriate for CFA to give rise to an effective pain reduction and to allow physiological changes in tendons, probably due to tissue membrane stabilization. It can be hypothesized that the mechanical changes produced by the CFAs were sufficient to control pain and maintain shoulder function for the follow-up period.

The goal of the treatment was that the subjects felt a better shoulder condition after the 10-days treatment, allowing to resume their normal work activities or hobbies, starting again to use and “stress” the shoulder, which was very often the dominant one.

The extremely positive finding that makes this observation even more promising is that the results were achieved with the only application of a natural product based on CFAs, without the use of physical therapies or use of corticosteroids. Moreover, the results obtained allows patients to return to their normal activities and the CS value were maintained without the administration of any treatment during the follow-up 35 days later. This observational study included a limited number of patients; integration of the data with a larger number of subjects in the future might be able to consolidate the results obtained. Moreover, the study lack of a control group. According with the experience of the authors, a “non-treatment” or an adhesive patch treatment only was not considered ethical and risked chronicizing tendinopathy. In the future, a study with another treatment as control, e.g. a physical therapy, could be designed to observe differences between two different approaches to shoulder tendinopathy. It would also be desirable to observe the ultrasound evolution of the tendinopathies for objectively assessing the reduction in oedema and the general improvement in tendon tissue.

## Conclusions

In conclusion, the application of CFA in patch formulation for 10 consecutive days in patients with shoulder tendinopathy was effective in reducing pain and resulted in a good recovery of joint function. The results achieved at day 10 were maintained for 25 days, until the follow-up visit at day. CFA-based patch seems to be a promising solution to improve pain and functionality in subject with shoulder tendinopathy.

## Data Availability

Raw data was provided in related files section.

## Abbreviations

CFA: Cetylated Fatty Acid
NSAIDs: Non-steroidal anti-inflammatory drugs
ROM: Renge of Motion
ADL: Activities of daily living
CS: Constant Score
